# The Gut Microbiota–Brain Axis during Aging, Mild Cognitive Impairment and Dementia: Role of Tau Protein, β-Amyloid and LPS in Serum and Curli Protein in Stool

**DOI:** 10.3390/nu15040932

**Published:** 2023-02-13

**Authors:** Mónica Sánchez-Tapia, Alberto Mimenza-Alvarado, Lizbeth Granados-Domínguez, Adriana Flores-López, Adriana López-Barradas, Victor Ortiz, Claudia Pérez-Cruz, Hilda Sánchez-Vidal, Julieta Hernández-Acosta, José Alberto Ávila-Funes, Martha Guevara-Cruz, Armando R. Tovar, Nimbe Torres

**Affiliations:** 1 Departamento de Fisiología de la Nutrición, Instituto Nacional de Ciencias Médicas y Nutrición Salvador Zubirán, Av. Vasco de Quiroga No. 15, Col. Belisario Domínguez, Sección XVI, Tlalpan, Mexico City 14080, Mexico; 2 Departamento de Geriatria, Instituto Nacional de Ciencias Médicas y Nutrición Salvador Zubirán, Mexico City 14080, Mexico; 3 Laboratorio de Neuroplasticidad y Neurodegeneración, Departamento de Farmacología, CINVESTAV, Mexico City 07360, Mexico

**Keywords:** dementia, intestinal microbiota, β-amyloid, tau protein, curli protein, LPS

## Abstract

Currently, there is an increasing number of people with mild cognitive (MCI) impairment and dementia (D). In the present work we studied the role of tau protein, β-amyloid, LPS (lipopolysaccharide), and curli protein of elderly adults with MCI or D and the contribution of gut microbiota. Four groups were studied: young subjects, healthy adults older than 60 years (A), elderly adults with MCI (MCI), and elderly adults with dementia (D). A preclinical study was conducted in old male Wistar rats to evaluate the impact of gut microbiota on curli protein abundance in feces and brain. The results showed that with increasing age, tau protein, β-amyloid, and LPS significantly increased in serum during MCI and D, and this was associated with an increase in the abundance of *E. coli* that synthesize the amyloid protein curli, that may promote the aggregation of amyloid proteins. Rats showed a clear increase in the abundance of curli protein in the brain during aging. Thus, cognitive impairment and dementia are in part due to an alteration in the gut microbiota–brain axis via increase in curli protein and LPS leading to an increase in tau and β-amyloid protein.

## 1. Introduction

Approximately 47 million people with dementia were reported in 2015 and it is estimated that by 2050 it will increase to 131 million, especially in low and middle-income countries, representing a global challenge for hospital and social care and having a strong economic impact in the twenty-first century [1,2]. Neurodegenerative diseases, particularly mild cognitive impairment (MCI) and dementia, are characterized by a decrease in cognitive skills, accompanied by neuroinflammation associated with an increase in intestinal permeability. This is in part due to changes in the diversity of the intestinal microbiome during aging, caused by the use of treatments for various types of diseases, physiological alterations, and an imbalanced diet, in addition to a progressive chronic proinflammatory response, known as inflammaging [3].

Cognition was originally thought to be exclusively regulated by the nervous system, but it is now becoming clear that many non-nervous system factors, particularly resident bacteria of the gastrointestinal tract, are involved in the regulation of cognitive function. Alzheimer’s disease (AD) is a progressive neurodegenerative syndrome, accounting for between 60% and 80% of all dementias [4] and is considered to be the most costly disease in the United States. AD is characterized by the formation of senile plaques (SP) formed by intracellular accumulation of β-amyloid peptide (Aβ) and neurofibrillary tangles formed by hyperphosphorylated tau protein aggregation [5,6]. Amyloid is defined as the aggregation of proteins into amyloid fibers found in many neurodegenerative disorders [7]. Functional amyloids are characterized as containing curli fimbria produced by *Escherichia coli*. On the other hand, the tau protein promotes the assembly of tubulin in microtubules in neurons; however, under pathological conditions, the tau protein self-polymerizes and aggregates in the form of neurofibrillary tangles (NTF) as found in Alzheimer’s disease [8]. Tau protein has been implicated in cognitive impairment during aging. Early findings now suggest that gut imbalance (dysbiosis) may contribute to age-related declines in cognitive function. Microbiota dysbiosis has been reported to trigger intestinal barrier dysfunction due to changes in the tight junctions and as a consequence, LPS, a component of the cell wall of Gram (−) bacteria [9] activates the toll-like receptor (TLR) which contributes to intestinal wall alterations by increasing leaky gut. It has been described that the intestinal microbiota interacts directly with the brain, allowing dynamic and bidirectional flow of information between the axis known as the microbiota–gut–brain, generating effects on cognition [10,11].

Furthermore, genetic predisposition is an important factor contributing to the development of AD, and some susceptibility genes are related to immune reaction, inflammation, cell migration, and lipid transport pathways [12]. The Apo E apolipoprotein is one of the most common susceptible genes with polymorphisms of three alleles: ε2 (protective allele), ε3 (neutral allele), and ε4 (high-risk allele) [13]. The most prevalent form of AD that constitutes almost 95% of all cases is associated with ApoE4, present in at least 20% of the population [14].

The timely detection of specific biomarkers plays an important role in the follow-up of AD patients or people at risk of developing the disease [8]. Therefore, the objective of the present work was to evaluate how changes in intestinal microbiota modify the formation of proteins associated with cognitive impairment and dementia in elderly people compared with healthy subjects.

## 2. Materials and Methods

### 2.1. Subjects

A cross-sectional study was conducted on volunteer subjects from central Mexico (Mexico City, Pachuca, Hidalgo, and Cuautla Morelos). This study was carried out in the Department of Nutrition Physiology of the National Institute of Medical Sciences and Nutrition, Salvador Zubirán (INCMNSZ). Two groups were included in this study: (1) a group of 35 healthy young subjects without comorbidities with a body mass index ≤ 25, (2) a group of 87 adults over 60 years of age. This group was subdivided into 3 groups, 42 adults older than 60 years without a diagnosis of cognitive impairment (A), a group of 32 subjects with mild cognitive impairment (MCI), and a group of 13 with dementia (D). The inclusion and exclusion criteria are shown in Supplementary Materials. Subjects in the study had a medical examination, anthropometric measurements, and a cognitive assessment.

A blood sample was taken to determine different biochemical parameters, lipid profile, glucose, lipopolysaccharide (LPS), tau protein, β-amyloid, branched chain amino acids (BCAA), and assessment of the presence of ApoE gene polymorphism. A stool sample was collected to determine the gut microbiota and the abundance of curli protein. A cognitive assessment consisted of the application of the neuropsychological test minimum mental state examination (MMSE) to determine the state of memory and establish the diagnosis of cognitive impairment in groups of adults older than 60 years (shown in Supplementary Materials). This study was carried out in accordance with the Declaration of Helsinki guidelines. The Ethics Committee approved all procedures involving humans of the Instituto Nacional de Ciencias Médicas y Nutrición Salvador Zubirán (No. 793, 2151 and 2968). All participants were informed about the scope and procedures of the study, and prior to any procedure, written informed consent was obtained formally.

### 2.2. Anthropometry and Body Composition

Weight and body composition including muscle mass and body fat were determined with a multifrequency bioelectrical impedance body composition analyzer (Inbody 720). Height was taken with an automatic stadimeter (biospace BSM370) with an error range of ±1 mm, for which patients were asked to be barefoot and placed in a Frankfort plane.

### 2.3. Blood Pressure

Blood pressure was measured with a digital baumanometer (Omron, Bannockburn, IL, USA, HEM-781INT) while the person was sitting with his right arm exposed. The measurement was taken three times; at intervals of three minutes, the first measurement was ignored, while the last two measurements were averaged to determine the systolic and diastolic pressure.

### 2.4. Biochemical Parameters

A blood sample was taken from the patient after 12 h of fasting. Blood was centrifuged at 3000 rpm for 10 min and serum was kept at a temperature of −80 °C until analysis. For biochemical parameters, a COBAS C111 clinical chemistry analyzer was used to quantify serum glucose, total cholesterol, HDL cholesterol, LDL cholesterol, and triglyceride concentrations. Branched chain amino acids were determined with the Ab83374 branched chain amino acid assay kit Abcam^®^.

### 2.5. Determination of ApoE Polymorphism

From a centrifuged whole blood sample with EDTA, leukocyte plaque was obtained and stored at −80 °C until processing. DNA isolation was carried out with a Qiagen DNA isolation kit. The presence of polymorphism of the ApoE gene was determined by Taqman probes rs429358 and rs7412, by PCR, and allelic discrimination. These genotypes showed the Hardy–Weinberg equilibrium. The ApoE polymorphism was determined by allelic discrimination using a Taqman SNP polymerase chain reaction end-point genotyping assay (ABI Prism 7900 HT Sequence Detection System; Applied Biosystems, Foster City, CA, USA).

### 2.6. Determination of Serum Tau and β Amyloid

Tau protein and amyloid were determined in serum samples using an enzyme-linked immunosorbent assay with the ab210972 kit—Human Tau SimpleStep ELISA^®^ and amyloid peptide (1–42) (human) Ab12030, Abcam.

### 2.7. Determination of LPS

The lipopolysaccharide was determined in serum samples using the CEB526Ge 96 enzyme-linked test immunosorbent assay for the lipid (LPS) kit according to the manufacturer’s instructions.

### 2.8. PCR End Point

To demonstrate the presence of the csgA gene, the major subunit of curli, we used 3 µL of total DNA extracted from feces at a concentration of 40 ng/µL. For this protocol we used 14.5 µL of free water of nucleases and 5.5 µL of high-fidelity DNA polymerase 2× KAPA HiFi HotStart ReadyMix and 1 µL of each primer at a concentration of 5 µM. The sequence of the primers used were: Forward: TCTGGCAGGTGTTGTTCCTC and Reverse: GGTCAGGGCTCAGATGACAG.

### 2.9. Western Blot of Curli Protein

Protein concentration from stool and brain samples was measured using the Bradford assay (Bio-Rad, Hercules, CA, USA). Thirty µg of protein was loaded and separated on a 10–12% SDS-PAGE gel and then transferred to a polyvinylidene difluoride (PVDF) membrane (Millipore Sigma). The membranes were blocked with 3% BSA in TBS Tween for 60 min at room temperature and incubated overnight at 4 °C with the primary antibody CsgA_ECO57 (major curlin subunit from *E. coli*) (Cusabio Technology LLC, Houston TX, USA.) at 1:3500. This antibody was previously validated [15]. After that, the membranes were incubated with the secondary antibody in the blocking buffer and blots were incubated with anti-rabbit (Abcam, AB6721) secondary antibody conjugated with horseradish peroxidase (1:20,000). GAPDH (1:40,000, ab181602) was used to normalize the data. Images were analyzed with ChemiDoc™ XRS + System Image Lab™ software (Bio-Rad, Hercules, CA, USA). The assays were performed three times using independent blots.

### 2.10. Fecal DNA Extraction

Fecal samples were placed in an OMR-200 Omnigene Gut Kit collection and DNA was extracted with the QIAMP DNA stool mini kit (Qiagen, Germantown, MD, USA) according to the manufacturer’s instructions.

### 2.11. Gut Microbiota Analysis by 16S rRNA Sequencing

From the total DNA of each subject, the V3 and V4 regions of the 16S rRNA ribosomal gene were amplified using specific forward (5′ TCGTCGGCAGCGTCAGATGTGTATAAGAGACAGCCTACGGGNGGCWGCAG 3′) and reverse primers (5′ GTCTCGTGGGCTCGGAGATGTGTATAAGAGACAGGACTACHVGGGTATCTAATCC 3′) containing the Illumina adapter overhang nucleotide sequences. Ampure XP beads were used to purify 16S V3–V4 amplicons, which were quantified by high-resolution capillary electrophoresis (Qiaxel, QIAGEN, Germany). The amplicon size was approximately 550 bp. Then, an index PCR was performed to attach the dual indices using a Nextera XT v2 kit. The amplicon was approximately 610 bp and the double-stranded DNA concentration was measured using a Qubit 3.0 fluorometer with a high-sensitivity kit. The final library of amplicons was pooled in equimolar concentrations. Sequencing was performed on the Illumina MiSeq platform (MiSeq Reagent Kit V.3, 600 cycles) at 15 pM with 20% Phyx infection according to the manufacturer’s instructions to generate paired end reads of 300 bases in length in each direction.

### 2.12. Animals

Male Wistar rats weighing 200 to 250 g were obtained from the Animal Facility of Instituto Nacional de Ciencias Médicas y Nutrición Salvador Zubirán. The animals were housed in individual stainless-steel cages at 22 °C with a 12:12 h light-dark cycle and allowed free access to water and a food diet. Serum and feces samples were collected at 2, 12, 18, and at the end of the study 24 months, where the rats were killed by cervical decapitation and the brain was immediately collected. This study was approved by the Animal Care Committee of the Instituto Nacional de Ciencias Médicas y Nutrición Salvador Zubirán (CICUAL: FNU1901-17-19/1) in accordance with international guidelines for the use of animals in research.

### 2.13. Statistical Analysis

Data were expressed as the mean ± standard deviation (S.D). Data were analyzed using one-way analysis of variance (ANOVA) followed by Tukey’s multiple comparison post hoc test. The association between two variables was calculated using the Spearman correlation. Significance was established at *p* < 0.05. Statistical analyzes were performed using Prism 9.0. Principal component analysis (PCA) was carried out to extract the important information from a multivariate data table. We used 32 variables including anthropometric, biochemical, cognitive variables, and bacterial species that were significantly more abundant in the lineal discriminatory analysis for the analysis, of PCA with the RStudio v1.2.1335 package. The database was standardized through centering and scale, followed by calculating the eigenvectors and eigenvalues from the covariance matrix or correlation matrix. Next, the eigenvalues were sorted in descending order and the K largest eigenvectors were selected (where K is the desired number of dimensions of the new feature subspace k ≤ 3). The projection matrix W was constructed from the selected K eigenvectors and finally the original dataset X was transformed through W to obtain a K-dimensional feature subspace Y. The graph was designed by factoextra in RStudio. Finally, we used a “corrplot” package in R studio to generate a graph of the correlation matrix known as Correlogram, to visualize the variables in a data set that are correlated the most. The correlation coefficients in the plot are colored based on the value. The blue circles represent positive correlations and the red circles negative correlations, while the diameter and intensity represent the r value in the coefficient of correlation.

## 3. Results

### 3.1. Anthropometric and Biochemical Parameters

When evaluating the body composition of the subjects, we found that the groups of adults older than 60 years (A), mild cognitive impairment (MCI), and dementia (D) showed significantly higher (19.5%) BMI than the young group (Y) (Table 1). The MCI or D groups had a higher percentage of body fat mass compared to groups A or Y by 4.5% and 13.8%, respectively (Table 1), and a similar pattern was observed in visceral fat (Table 1). Regarding skeletal muscle mass (SMM), notably, the D group showed 34.9% less SMM compared to the other groups (Table 1). The MCI or D groups showed significantly 35.5% and 42.1% higher serum glucose levels (Table 1) than the Y group. The prevalence of type 2 diabetes in the MCI and D groups was 25 and 15.4%, respectively, while in the A group it was 2.4% (Table 1). Regarding biochemical parameters, the A group showed a significant increase (73%) in serum triglycerides compared with the Y group, but there were no changes in serum total cholesterol in the groups (Table 1). However, LDL cholesterol increased significantly by 40.2% and 30.3% in the A and MCI groups compared to the Y group (Table 1). HDL cholesterol showed a significant decrease in the MCI and D groups in men by 34%, while in women the decrease was 31.5% and 49% in the MCI and D group, respectively, compared to the Y group (Table 1).

### 3.2. Inflammation and Neuronal Biomarkers

**Figure 1:**
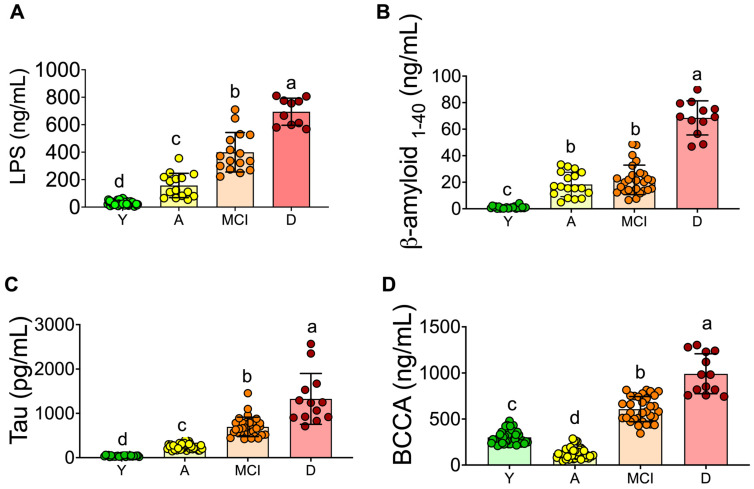
Serum biomarkers associated with inflammation and neurodegeneration in serum of young and aging adults. Lipopolysaccharide (**A**). Tau protein (**B**), β-amyloid protein 1–40 (**C**), and total branched-chain amino acids (**D**) in serum. Raw data and mean are presented ± standard deviation. Y = young, A = aging, MCI = mild cognitive impairment, D = dementia, a > b > c > d, significance *p* < 0.05.

To study the potential circulating biomarkers associated with neuronal damage, serum LPS, tau protein, and β-amyloid were measured in all groups. Figure 1A shows that the LPS concentration was very low in the Y group (26.4 ng/mL). In the LPS A group, the concentration was 157 ng/mL, five times higher than in the Y group. Interestingly, the concentration of LPS in the MCI and D groups was 399 ng/mL (14 times) and 694 ng/mL (25 times), respectively, indicating the presence of low-grade inflammation during aging.

β-amyloid and tau have been implicated in the formation of insoluble plaques and tangles as the main toxic agents in Alzheimer’s disease (AD). Interestingly, serum β-amyloid was significantly higher in the A and MCI groups compared to the Y group; however, the D group showed the highest concentration of this peptide with a concentration of 68.5 ng/mL, 2.7 times higher than the A group and 68 times higher than the Y group (Figure 1B). With respect to the tau protein, a similar pattern was observed. The group with the highest tau concentration was the D group with 1326 pg/mL, 35 times higher than the Y group (Figure 1C). Diabetes has been described as resulting in increased blood–brain barrier (BBB) permeability and amyloid deposition [16], and since some of the subjects in the MCI or D groups showed type 2 diabetes, we included a group of subjects with type 2 diabetes. The serum tau protein in these subjects showed a concentration of 927 pg/mL, values significantly higher than the MCI group with 623 pg/mL, indicating that the presence of type 2 diabetes could be a risk for developing MCI.

Serum branched chain amino acids (BCAA) have been associated with type 2 diabetes and Alzheimer’s disease [16], therefore we measured these amino acids in all groups and found that subjects in the MCI and D groups had increased concentration of these amino acids by 102% and 229%, respectively, compared to the Y group, Figure 1D. Interestingly, the MCI and D groups showed 25% and 15.4% of the prevalence of type 2 diabetes, in addition to the fact that these groups showed as overweight and had low skeletal muscle mass. However, the BCAA concentration in healthy aging rats (18 and 24 months) was significantly lower than in healthy adult rats (12 months) with normal concentration of glucose, indicating that the presence of metabolic diseases, particularly type 2 diabetes is associated with an increase in the circulating levels of BCAA [17].

### 3.3. The Gut Microbiota throughout Life in Humans

**Figure 2:**
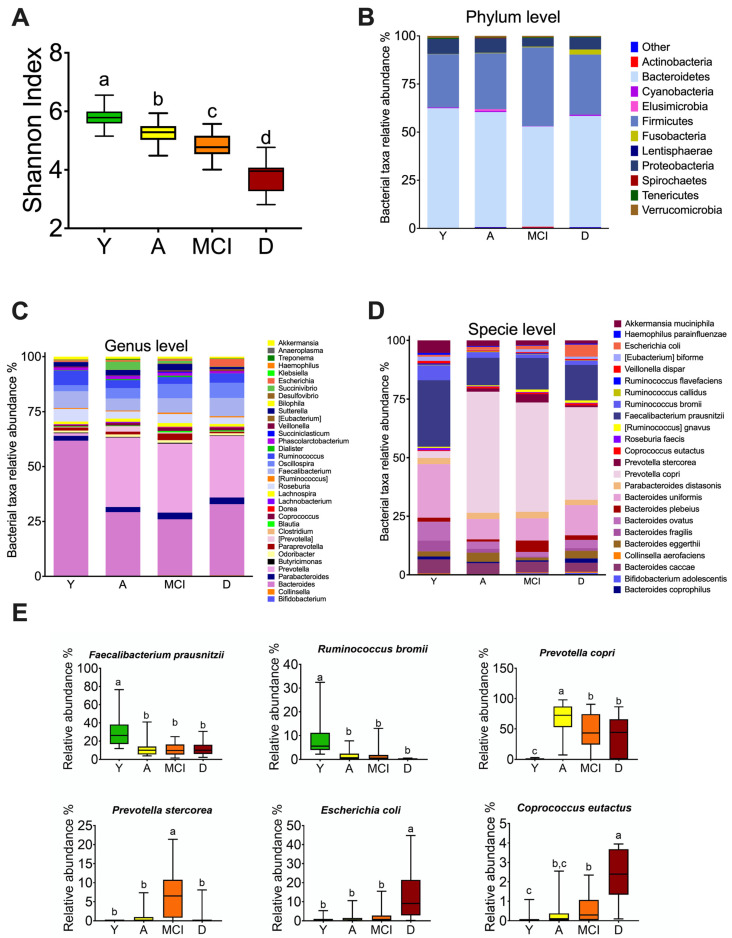
Changes in the gut microbiota profile associated with mild cognitive impairment and dementia in humans. Shannon index (**A**). Relative abundance of the intestinal microbiota at the level of phylum (**B**), genus (**C**), and species (**D**). Operational taxonomic units at the species level with significant changes among all groups (**E**). Y = young, A = aging, MCI = mild cognitive impairment, D = dementia, a > b > c > d, significance *p* < 0.05.

To investigate whether the changes observed in the gut microbiota were associated with age or the presence of MCI or dementia, we evaluated alpha diversity, as well as taxonomic assignment to different levels. The reads per sample were 43,587 ± 3928 with a 97% similarity threshold. Thus, 99.9%, 82.4%, and 43.6% of the reads were assigned to the phylum, genus, and specie level, respectively. Bacterial diversity decreases with increasing age, the group of adults >60 years without comorbidities had a lower Shannon index compared to the young group; however, the groups with MCI and D showed an even lower Shannon index (Figure 2A). At phylum level, the MCI group showed an increase in Firmicutes and the D group showed a significant increase in the Fusobacteria phylum (Figure 2B). At the genus level, the A, MCI, and D groups showed an increase in *Prevotella* and a decrease in the *Bacteroides* genus with respect to the young group. Furthermore, group A showed an increase in *Succinivibrio*, the group with MCI showed an increase in *Paraprevotella*, and group D showed an increase in the *Escherichia* genus (Figure 2C). Interestingly, at the species level, there was a significant increase in *Prevotella copri* in the A, MCI, and D groups compared to the Y group (Figure 2D). To determine the significant changes at the species level, we performed a linear discriminant analysis and found a significant increase in *Faecalibactrium prausnitzii* and *Ruminococcus bromii* in the Y group. The A, MCI, and D groups had a significant increase in *Prevotella copri*, and the MCI group showed an increase in *Prevotella stercorea*. Interestingly, the D group showed a significant increase in *Escherichia coli* and *Coprococcus eutactus* (Figure 2E).

**Figure 3:**
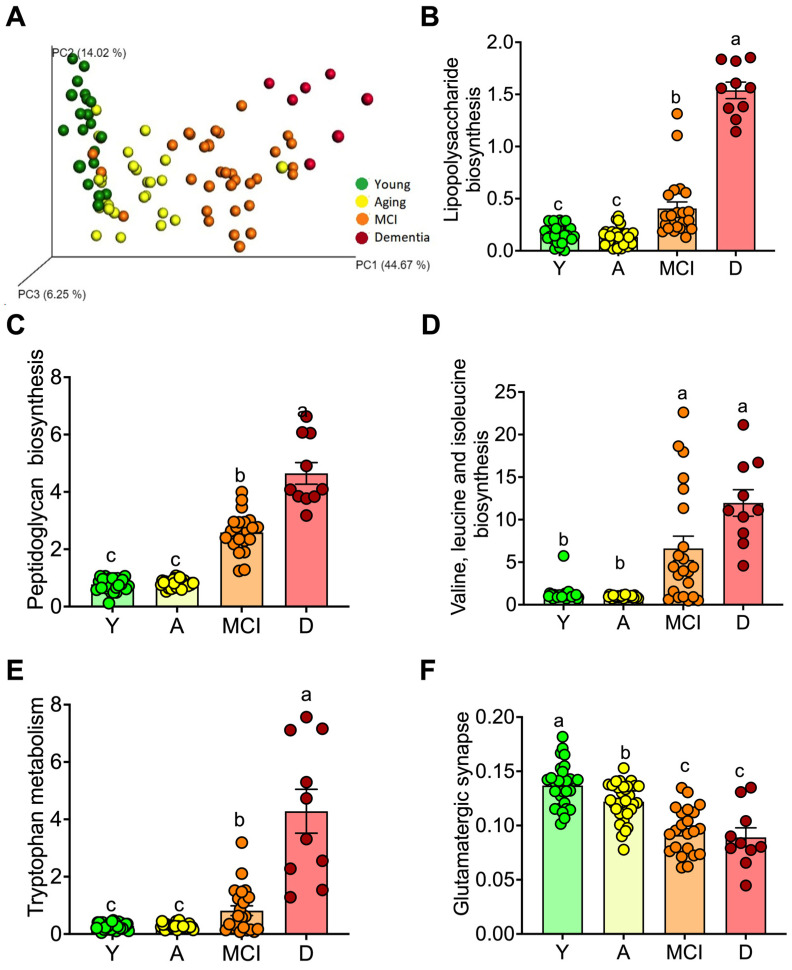
Predictive functional profiling of gut microbiota (Picrust) in young and aging adults. Principal component analysis of prediction of the functional profile of gut microbiota (**A**). Main metabolic pathways modified: lipopolysaccharide biosynthesis (**B**), peptidoglycan biosynthesis (**C**), branched amino acid biosynthesis (**D**), tryptophan metabolism (**E**), and glutamatergic synapse (**F**) in young, and in healthy aging subjects, aging subjects with mild cognitive impairment (MCI), and dementia (D). Raw data and mean are presented ± standard error. Y = young, A = aging, MCI = mild cognitive impairment, D = dementia, a > b > c, significance *p* < 0.05.

Next, we assessed the metabolic pathways activated in the gut microbiota by age or the presence of MCI or dementia by using the PICRUSt analysis (Phylogenetic Investigation of Communities by Reconstruction of Unobserved States) (Figure 3A) [18]. The gut microbiota of subjects indicated that the biosynthesis of LPS was highest in D group followed by MCI group compared to Y and A groups (Figure 3B). This pattern was also observed for peptidoglycan biosynthesis (Figure 3C), biosynthesis of BCAA (Figure 3D), and tryptophan metabolism (Figure 3E), whereas there was a significant decrease in the glutamatergic synapse system (Figure 3F). The results of this predictive analysis agree with the circulating levels of LPS and BCAA. In addition, the increase in peptidoglycan biosynthesis indicated that also Gram (+) bacteria could be involved in the increase of proinflammatory cytokines. The increase in tryptophan metabolism was mainly associated with an increase in the catabolism of tryptophan by the gut microbiota that potentially may alter serotonin synthesis [19]. The decrease in the glutamatergic synapse system suggests a dysregulation of neuronal communication [20].

### 3.4. Curli Protein during Different Periods of Life in Humans

**Figure 4:**
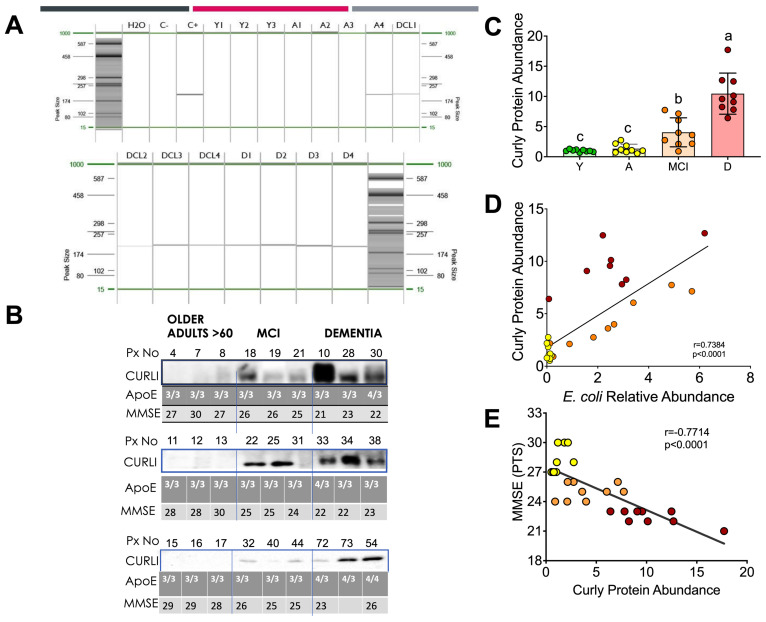
Association between the curli protein, polymorphism in ApoE and cognitive classification. PCR (**A**) and Western blot analysis of curli protein in human fecal samples, ApoE genotyping, and MMSE scoring (**B**). Densitometric analysis of curli protein in human fecal samples (**C**). Correlation analysis between the relative abundance of curli protein and *Escherichia coli* relative abundance (**D**) and the MMSE score (**E**). Raw data and mean are presented ± standard error. Y = young, A = aging, MCI = mild cognitive impairment, D = dementia, a > b > c, significance *p* < 0.05. Statistical significance was assessed by Spearman r. *p* < 0.05.

Curli proteins are expressed by certain intestinal bacteria such as *Escherichia coli*. Since we found an increase in *E. coli* in the intestinal microbiota of the D group, we studied the abundance of this protein in the feces of the groups at different stages of life. Interestingly, all subjects with dementia who tested for the presence of curli protein in feces by PCR (Figure 4A) or Western blot (Figure 4B) showed the presence of curli protein. The abundance of curli protein was associated with the relative abundance of *E.coli* found in the gut microbiota. Interestingly, subjects with mild cognitive impairment also showed the presence of curli protein, but to a lesser extent than the D group. The abundance of curli protein in the D and MCI groups was nine and four times higher than in the Y group (Figure 4C). There was a significant positive correlation between curli protein abundance and *E. coli* (*p* < 0.001), Figure 4D.

Since other factors such as the presence of some genetic variants in the ApoE gene, mainly ApoE 4, are associated with the development of Alzheimer’s disease, this polymorphism was measured in these subjects. Interestingly, all subjects with the presence of the Apo E4 polymorphism had the presence of the curli protein (Figure 4B). To identify cognitive impairment in subjects in the MCI and D groups, a mini mental state examination (MMSE) was performed, indicating that the D group showed the lowest score followed by the MCI group, while the A group showed an adequate score in the MMSE test. In fact, there was a significant inverse correlation (*p* < 0.001) between curli protein abundance and the MMSE score (Figure 4E).

### 3.5. Gut Microbiota during Different Periods of Life in Rats

**Figure 5:**
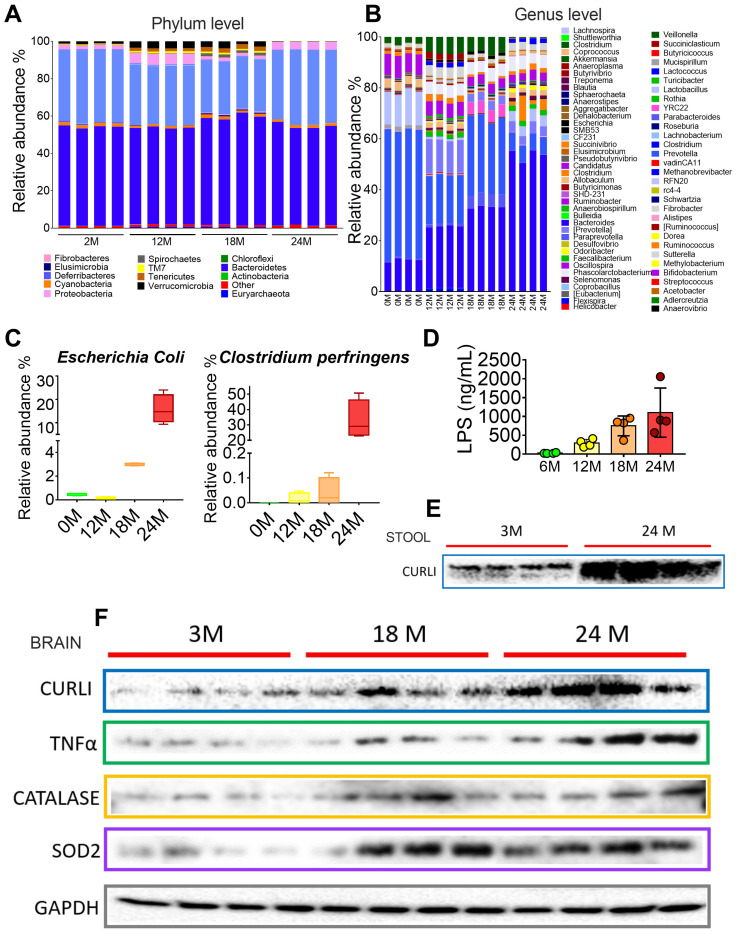
Aging modifies the intestinal microbial profile and increases inflammation in rats. Relative abundance of the intestinal microbiota at the level of phylum (**A**), genus (**B**), and species (**C**), and serum lipopolysaccharide (**D**). Abundance of curli protein in feces (**E**). Western blot analysis of markers of inflammation and oxidative stress in the brain of rats at different ages (**F**).

Since we were unable to measure curli protein in the brain of subjects in the different groups, we proceeded to measure curli protein in the brains of rats at different ages. To study whether the curli protein was present in the brain and stool of rats during aging, we studied the intestinal microbiota of rats at different periods of life at 2, 12, 18, and 24 months. The intestinal microbiota revealed that, at the phylum level, 24 month old rats had a significant increase in Tenericutes and a decrease in Deferribacteres and Verrucomicrobia (Figure 5A). At the genus level, there was an increase over time in the genus *Bacteroides*, and a significant increase was also observed at 24 months in the genus *Dorea, Blautia,* and *Escherichia*, as well as a considerable decrease in the genus *Akkermansia* (Figure 5B). At the species level, there was a significant increase at 18 and 24 months for *Escherichia coli*, and particularly at 24 months, there was an increase for *Clostridium perfringens* (Figure 5C). In particular, curli protein in the stools showed a significant increase at 24 months (Figure 5E). Finally, there was a gradual increase in brain curli protein at 18 and 24 months of age of 1.4 and 3.8 times compared to young rats, which was associated with neuroinflammation, since *E.coli* is a Gram (−) bacterium that increases LPS as a marker of low-grade inflammation. LPS gradually increased over time (12, 18, and 24 months), with values of 11, 30, and 45 times higher than those presented in 2-month-old rats (Figure 5D). LPS, in turn, could activate the production of TNFα pro-inflammatory cytokine 1.7 and 3.9 times at 18 and 24 months compared to young rats. TNFα, which is released by activated astrocytes and microglia, can exacerbate inflammation, and promote ROS release. ROS are converted to H_2_O_2_ by superoxide dismutase 2 (SOD2) [21]. As can be seen in Figure 5F, the abundance of SOD2 increased by 1.9 and 3.4 times, while TNFα selectively affected Nrf2 dependent antioxidant regulation by reducing brain Nrf2 by 44 and 70% compared to the young group (Figure 5F). These results indicate that enhanced levels of curli and LPS may be associated with neuroinflammation, which in turn may promotes the formation of amyloid plaques.

**Figure 6:**
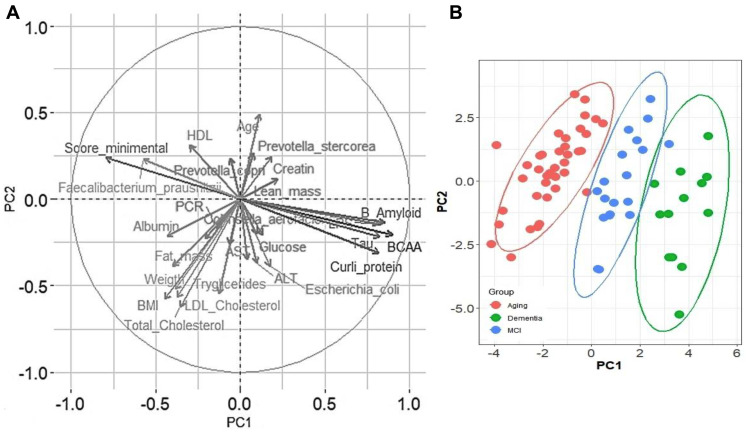
Association among biochemical parameters, gut microbiota, and cognitive evaluation. Variables associated with the distribution of groups and their contribution to PCA (**A**) Distribution of older adults according to their cognitive classification in the principal component analysis (**B**).

**Figure 7:**
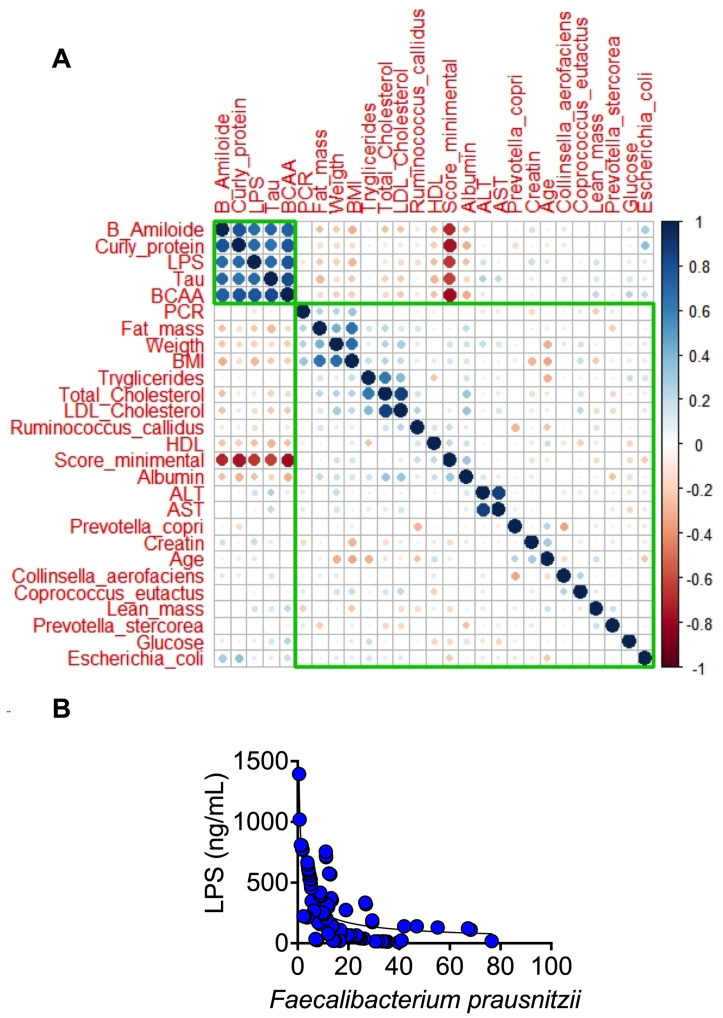
Analysis of multiple correlations between variables measured in the study (**A**). Correlation analysis between the serum concentration of LPS and the abundance of *Faecalibacterium prausnitzii* (**B**). Statistical significance was assessed by Spearman r. *p* < 0.05.

To evaluate the contribution of the different variables in the development of MCI or dementia, a principal component analysis was performed with a total of 32 variables, including anthropometric, biochemical, cognitive variables, and bacteria that were significantly more abundant in the lineal discriminatory analysis (LDA), to know if there was a pattern among the studied groups and which of these variables could have a greater importance in the distribution of the groups. As a result of the sedimentation graph, it was observed that with three components, more than 80% of the variance of the data was explained. When analyzing the data on how the samples were distributed, we visualized the presence of three groups, from left to right, A, MCI, and D groups (Figure 6B). As shown in the load graph (Figure 6A), the A group was characterized by having a higher score on the minimental test, a higher HDL serum concentration, and a significant increase in the abundance of *Faecalibacterium prausnitzii*, while the D group showed an increase in serum βamyloid protein, tau protein, LPS, and curli protein in feces, while the MCI group showed an increase in fat mass, weight, body mass index, and serum triglycerides. On the other hand, when a correlation matrix was carried out to analyze the relationship between each pair of numeric values, we observed that there was a cluster of variables that correlated positively with each other, these were β amyloid, curli, tau proteins, LPS, and BCAA. These variables are interestingly negatively correlated with the cognitive evaluation of the minimental test. On the other hand, *Escherichia coli* was notably positively correlated with curli and β amyloid proteins, while serum HDL concentration did so negatively (Figure 7A). Finally, it is important to note that as LPS increased, the relative abundance of *Faecalibacterium prausnitzii* decreased, indicating that *F. Prausnitzii* has anti-inflammatory properties (Figure 7B).

## 4. Discussion

Between 2000 and 2050, the proportion of the world’s population aged 60 years and older will double from 11% to 22% [22]. Therefore, it will be necessary to develop an integrated approach to public health actions to ensure healthy lives and promote well-being. The present work as well as previous studies have shown that during the aging period, a progressive chronic proinflammatory response, known as inflammaging develops [23]. In younger people, inflammation is a fundamental response to cope with internal and external damaging agents; however, in later life, it can be detrimental, contributing to the development of various age-related chronic diseases such as type 2 diabetes and Alzheimer’s disease, as we demonstrated in the aging groups in this study. Our results showed that the dementia group had the highest serum LPS concentration that reflects low-grade chronic inflammation compared to the young, adult and even mild cognitive impairment groups. These results are in part due to the aging process, since in our preclinical study, aging rats (24 months old) showed the highest concentration of LPS, a molecule derived from Gram (−) bacteria of the gut microbiota even though the rats had no metabolic diseases or medication consumption. Interestingly, in the group with dementia, serum LPS levels were 25.3 times higher than those of healthy controls, indicating that disturbances along the brain–gut–microbiota axis may significantly contribute to the pathogenesis of neurodegenerative diseases due to alterations in the gut microbiota composition, that induce increased permeability of the gut barrier and immune activation leading to systemic inflammation [24].

Furthermore, during aging, mitochondria produce a toxic byproduct of ATP synthesis, reactive oxygen species (ROS). It is known that TNFα stimulates the production of ROS that are converted to H_2_O_2_ by superoxide dismutase 2 (SOD2) in the brain. We found that TNFα protein abundance increased with aging as well as the abundance of brain SOD2 and catalase; catalase converts H_2_O_2_ to H_2_O, reducing the damaging effects of ROS.

The most prominent characteristics during dementia are the accumulation of plaques of amyloid beta peptides (Aβ). Aβ induce the hyperphosphorylation of tau which aggregates into neurofilaments tangles that correlate well with the progressive decline of cognitive functions. Interestingly, we found that Aβ and tau protein were significantly higher in blood samples in the groups with mild cognitive impairment and dementia compared to the adult group, indicating that tau protein and Aβ could be used as potential biomarkers of dementia, as there was a significant inverse correlation between LPS, Aβ, and tau protein with neurological evaluation (Figure 7A). Besides these biomarkers, there is evidence that genetic susceptibilities are also risk factors for developing neurological diseases (NDs). Apolipoprotein E (ApoE) gen is one of the most common susceptibility genes with three allele polymorphisms (ε2, ε3, and ε4), in which ε4 is a high-risk allele. Although aging, family history, and susceptibility genes have been considered the most important factors, environmental factors [25] experienced throughout life also impact the onset, progression, and severity of NDs. As can be seen in Figure 4B, not all dementia patients showed the presence of ApoEε4, however, all dementia patients showed the presence of curli protein, produced by *E. coli* of the intestinal gut microbiota, indicating that environmentally induced alterations in the intestinal microbiome may contribute in part to the regulation of brain function through the microbiota–gut–brain axis, as has been demonstrated in the pathogenesis of other neurological disorders. In fact, our study of old rats without medications or metabolic diseases also showed an increase in curli protein in the stool and brain, *E. coli*, and LPS, indicating that aging is commonly accompanied by chronic inflammation and increased intestinal permeability suggesting strong evidence of a microbiota–gut–brain axis.

Considering that changes in the gut microbiota throughout life, associated with pro- and anti-inflammatory balance as well as with immune function, affect the gut-brain axis, we found a decrease in intestinal bacterial diversity of the gut microbiota with age, particularly a significant decrease in *Faecalibacterium prausnitzii* in adults over 60 years of age. This bacterium has been considered to have anti-inflammatory properties and to be crucial for gut homeostasis [26]. There was a significant inverse correlation between *F. prausnitzii* and serum LPS concentration, indicating the important role of this bacterium in the regulation of inflammation (Figure 7B). *F. prausnitzii* is a Gram-positive bacterium belonging to the family Ruminococcaceae that has been reported to decrease in Italian, Swedish, Dutch, French, and Austrian elderly people [16]. Thus, we have to consider that gut microbiota could play an important role in the development of neurological diseases, since bacteria can produce functional extracellular amyloid proteins [27]. Therefore, it is necessary to carry out studies in larger populations to see if the results reported in this work can be applied to other populations. Bacterial amyloid proteins are involved in biofilm formation and help bacteria with invasion. The main bacterial amyloid protein is curli made by *E.coli,* indicating that the precursor factor for amyloid proteins [27] comes from the gut priming of immune cells to respond to these amyloids in the gut and triggering enhanced responses to neuronal amyloids in the brain. In summary, increase in BCAA, serum β-amyloid, LPS, tau, curli protein, *E.coli,* and a low minimental score were significantly associated with dementia followed by mild cognitive impairment (Figure 6A,B), indicating that these parameters can be used as biomarkers to detect mild cognitive impairment or dementia. It is known that metabolic diseases such as diabetes can result in increased blood–brain barrier permeability that potentially, further progresses to Alzheimer’s disease with the accumulation of amyloid beta peptides. More research is needed to determine whether bacterial amyloids of the gut microbiome may act directly or indirectly to increase brain Abeta peptide aggregation. Thus, the gut microbiome can modulate circulating biochemicals, some of which may interact directly with amyloids to promote aggregation in neurons and propagation from the periphery to the brain [28].

## 5. Conclusions

We demonstrated that dysbiosis in gut microbiota increased serum levels of tau protein, β-amyloid, and LPS as well as stool curli protein during mild cognitive impairment and dementia, these results were associated with an imbalance in the intestinal microbiota that increased *E.coli* abundance, which produces the amyloid curli protein responsible for the formation of neurofibrillary tangles. These results were associated with an increase in curli protein in the brain and an increase in oxidative stress during aging of rats. Therefore, these markers could be used for early detection of the presence of cognitive impairment, indicating an important link between the gut and brain. More studies are needed to confirm the assessment of these proteins as potential biomarkers for the early detection of mild cognitive impairment and dementia.

## Figures and Tables

**Table 1:** Anthropometric and biochemical variables in healthy young subjects and older adults with mild cognitive impairment (MCI) and dementia (D).

	Health	Aging	MCI	Dementia	*p*
	*n* = 29	*n* = 42	*n* = 32	*n* = 13	
Biochemical					
Glucose, mg/dL	85.3 ± 10.5	98.2 ± 14.3	116 ± 57.3	121 ± 58.6	0.01
Triglycerides, mg/dL	89.9 ± 33	150 ± 104	143 ± 103	150 ± 42	0.02
Total cholesterol, mg/dL	167 ± 35.1	192 ± 44	181 ± 43	174 ± 34	0.11
Demographics					
Age, years	31.1 ± 9.7	66.2 ± 7.0	74.3 ± 7.80	68.6 ± 7.0	0.0001
Female sex, *n* (%)	19 (65.5%)	28 (66.6%)	16 (50%)	11 (84.6%)	0.15
Education, years	15.2 ± 2.10	12.9 ± 4.90	11.0 ± 5.18	11.5 ± 5.0	0.031
Body mass index, kg/m^2^	22.6 ± 1.72	28.2 ± 4.43	27.7 ± 4.31	25.7 ± 3.8	0.001
Body fat mass, %	29.2 ± 8.4	33.2 ± 9.4	35.7 ± 8.5	45.0 ± 5.9	0.03
Visceral fat mass, cm^2^	68.3 ± 18.9	118 ± 27.0	95.9 ± 24.1	130 ± 34.1	0.0001
Skeletal muscle mass, kg	43.8 ± 10.5	22.5 ± 6.0	21.2 ± 4.5	14.6 ± 1.9	0.0001
Hypertension, *n* (%)		17 (40.5%)	15 (46.9%)	4 (30.8%)	0.777
Diabetes mellitus, *n* (%)		1 (2.4%)	8 (25.0%)	2 (15.4%)	0.033
Dyslipidemia, *n* (%)		10 (23.8%)	10 (31.3%)	2 (15.4%)	0.881
CKD, *n* (%)		1 (2.4%)	1 (3.1%)	0	0.871
IHD, *n* (%)		1 (2.4%)	3 (9.4%)	1 (7.7%)	0.424
Smoking habit, *n* (%)		9 (21.4%)	9 (28.1%)	3 (23.1%)	0.671
Alcohol consumption, *n* (%)		8 (19.0%)	6 (18.8%)	2 (15.4%)	0.870

Data are presented as mean ± standard deviation or number of patients (%). One-way ANOVA and chi-square test were used. Abbreviations: CKD, chronic kidney disease; IHD, ischemic heart disease; ApoE, apolipoprotein E; MCI mild cognitive impairment; ND (Not determined).

## Data Availability

Not applicable.

## Supplementary Materials

The following supporting information can be downloaded at: https://www.mdpi.com/article/10.3390/nu15040932/s1.
