# Supplementary material for: The Gut Microbiota–Brain Axis during Aging, Mild Cognitive Impairment and Dementia: Role of Tau Protein, β-Amyloid and LPS in Serum and Curli Protein in Stool
Source: Nutrients. 2023 Feb 13;15(4):932. doi: 10.3390/nu15040932 (PMC9961602; doi:10.3390/nu15040932)
Supplement: Supplementary file 1 [file nutrients-15-00932-s001.zip › nutrients-2195674-supplementary.pdf]

Criteria for the healthy young group were men and women 18 to 48 years of age, with a BMI of 18.5 to 24.99 kg/m^2, with the following exclusion criteria: presence of a disease and/or administration of any medication. In the group of people over 60 years of age, the inclusion criteria were men or women over 60 years of age, with adequate visual and auditory acuity to perform neuropsychological tests. Exclusion criteria were dysthyroid (hypo or hyperthyroidism) without treatment in the last six months, major depression without treatment, patients with uncontrolled type 2 diabetes or hypertension greater than 140/90 mmHg, dyslipidemias, cancer, heart disease, renal or pulmonary degenerative diseases in advanced stages. Other exclusion criteria were the use of acetylcholinesterase inhibitor drugs (donepezil, rivastigmine, galantamine) or memantine, other neurological or psychiatric disorders, including epilepsy, tumors, substance abuse, delirium or aphasia, the consumption of dietary supplements, antibiotic treatment two months before the study or laxative treatment one month before. Furthermore, subjects with therapy with probiotic/prebiotic/symbiotic foods and fiber-rich foods (>15 g of fiber), patients with a functional digestive disorder (constipation, diarrhea, dyspepsia, or functional abdominal distension determined by a questionnaire based on Rome III classification), inflammatory bowel disease, irritable bowel syndrome, or other chronic gastrointestinal diseases, and patients with major abdominal surgery were excluded.

Elimination criteria included whether the collection of stool samples for the microbiota was insufficient and the DNA quality obtained was inadequate or degraded for the determination of the gut microbiota.

The patients underwent a medical examination that consisted of an interrogation of current symptoms they presented in order to rule out uncontrolled chronic diseases or neurological and psychiatric diseases that can be confused with cognitive impairment, such as epilepsy, tumors, substance abuse, delirium, or aphasia

Neurological assessment. All participants over 60 years of age underwent a neurological assessment evaluation that included the application of the minimum mental state examination (MMSE). Participants with a clinical presumption of cognitive impairment were also evaluated using the Brief Neuropsychological Battery (Neuropsi), which allowed a wide spectrum of cognitive functions to be evaluated, including orientation, attention, visuospatial skills, memory, language, literacy, and executive functioning in the Mexican population [16]. Furthermore, the degree of functionality in basic and instrumented activities was determined through the Katz index and the Lawton-Brody index.

The diagnosis of dementia was made following the criteria of the International Statistical Classification of Diseases (ICD) in its tenth version [17] and the recommendations of the National Institute of Aging and the Alzheimer's Association [18]. Participants with dementia scored on MMSE between 21 and 23 points. The diagnosis of mild cognitive impairment was based on the criteria proposed by Petersen [19], which include memory complaints corroborated by an informant, deficits in at least one cognitive domain measured by the Neuropsi battery, considering deficits that score below 1.5 standard deviations, together with the conservation of activities of daily living; This group of participants presented MMSE scores between 24 and 26 points. Participants classified as adults over 60 years of age obtained scores on MMSE between 27 and 30, neither participants nor informants (partners or dependents) reported complaints of memory or difficulties in carrying out their daily activities autonomously.
